# Cancer patterns among children of Turkish descent in Germany: A study at the German Childhood Cancer Registry

**DOI:** 10.1186/1471-2458-8-152

**Published:** 2008-05-07

**Authors:** Jacob Spallek, Claudia Spix, Hajo Zeeb, Peter Kaatsch, Oliver Razum

**Affiliations:** 1 Dept. of Epidemiology & International Public Health, School of Public Health, Bielefeld University. P.O. Box 10 01 31, 33501 Bielefeld, Germany; 2 German Childhood Cancer Registry (GCCR), Institute for Medical Biostatistics, Epidemiology and Informatics (IMBEI), Obere Zahlbacher Strasse 69, 55131 Mainz, Germany; 3 Department of Epidemiology, Institute of Medical Biostatistics, Epidemiology and Informatics (IMBEI), Johannes Gutenberg – University Mainz, Obere Zahlbacher Str. 69, 55131 Mainz, Germany

## Abstract

**Background:**

Cancer risks of migrants might differ from risks of the indigenous population due to differences in socioeconomic status, life style, or genetic factors. The aim of this study was to investigate cancer patterns among children of Turkish descent in Germany.

**Methods:**

We identified cases with Turkish names (as a proxy of Turkish descent) among the 37,259 cases of childhood cancer registered in the German Childhood Cancer Registry (GCCR) during 1980–2005. As it is not possible to obtain reference population data for children of Turkish descent, the distribution of cancer diagnoses was compared between cases of Turkish descent and all remaining (mainly German) cases in the registry, using proportional cancer incidence ratios (PCIRs).

**Results:**

The overall distribution of cancer diagnoses was similar in the two groups. The PCIRs in three diagnosis groups were increased for cases of Turkish descent: acute non-lymphocytic leukaemia (PCIR 1.23; CI (95%) 1.02–1.47), Hodgkin's disease (1.34; 1.13–1.59) and Non-Hodgkin/Burkitt lymphoma (1.19; 1.02–1.39). Age, sex, and period of diagnosis showed no influence on the distribution of diagnoses.

**Conclusion:**

No major differences were found in cancer patterns among cases of Turkish descent compared to all other cases in the GCCR. Slightly higher proportions of systemic malignant diseases indicate that analytical studies involving migrants may help investigating the causes of such cancers.

## Background

The population of migrants in Germany comprises more than 7 million people with a Non-German nationality, and about 8 million national Germans with a migrant background. Thus, about 15 million or 19% of the resident German population have a migration background. This figure includes former guest workers, who came to Germany in the 70ies and 80ies mostly from south east Europe, refugees from all over the world and naturalised or ethnic German migrants from the former Soviet Union [1]. The number of migrant children is increasing. About 1 million children aged 0–15 years currently have a Non-German citizenship. The largest group of children with a Non-German citizenship in Germany are migrants from Turkey, comprising about 25% [2]. On the other hand, the number of naturalised children of Turkish origin with German nationality is increasing.

So far knowledge about the cancer risk of children with a migrant background in Germany is scarce. However, the available evidence indicates that the question is worth studying. There is evidence of world wide geographic variation in the incidence of childhood cancer [3]. In the Globocan 2002 database [4], the incidence rates of children in Turkey and Germany show differences: in Turkey, children aged 0–14 years have a higher incidence of Hodgkin's disease and non-Hodgkin lymphoma and a lower incidence of leukaemia and brain tumours compared to German children. It is unclear whether this difference is due to methodological differences (different data quality, different ways of data collection, different completeness and representativeness) or is indeed an expression of real differences in incidence. In Sweden, Hemminki & Li found increased cancer risks for migrant children of different origins and for different diagnoses compared to Swedish children, e.g. for lymphoma among children with Turkish parents [5].

In Germany, most routine health data do not contain valid and complete information on migrant status. Information on citizenship is unhelpful because it excludes the large and increasing number of migrants with German nationality. For example, the GCCR routinely collects no information relevant to migration status of registered cases. The underlying reasons are practical – the notifying physician or pathologist is often not aware of the migrant status of the diagnosed patient – rather than ethical. There is no legal requirement concerning in – or exclusion of indices of migrant status in a case report to the cancer registry. In consequence, migrant-specific analyses of cancer patterns or cancer risk are not available. This study, for the first time, presents data about childhood cancer for children with Turkish names in Germany.

## Methods

In Germany, all cases of childhood cancer are registered in a central register, the German Childhood Cancer Registry (GCCR) in Mainz. Between 1980–2005, 37,259 cancer cases below 15 years of age have been registered with open names based on parental consent. The completeness of registration and the quality of data are high and comply with international standards. More than 95% of all childhood cancer cases are registered; only for brain tumours the proportion is slightly lower [6]. This high completeness was reached in 1987 for the area of the former Federal Republic of Germany and shortly after the German reunification in 1990 for the area of the former German Democratic Republic. While the GCCR data has been used for a number of epidemiologic analyses, so far no investigations stratified for migration background or ethnicity have been possible. As previously explained, In the GCCR no data about citizenship, ethnic background or place of birth have routinely been collected in the past. Therefore, little is known about the cancer risk of migrant children in general and of children of Turkish origin in Germany in particular.

We applied a recently developed name-based approach to identify children of Turkish origin in the data base of the GCCR, which has been successfully used in previous studies [7,8]. The name algorithm is based on the high specificity of Turkish names as compared to German, central European, Arabic, or Asian names. This high specificity is the result of a name reform in Turkey in the 1930s when all inhabitants of Turkey had to adopt family names with a meaning in the Turkish language. Consequently, a person with a Turkish name has a very high probability of being of Turkish descent.

The name algorithm uses a list of more than 13,000 known Turkish family and first names to identify Turkish persons in the data base. It has an automatic part and a manual part. In the first automatic part, persons with names, which are definitely Turkish, were identified. Persons with names that are possibly Turkish or so called "doublets", i.e. rare names common in the German and Turkish language, were assessed in a second manual step by a native Turkish person, using all other available information (names of the parents, place of birth etc.) as far as available from patient records. To examine the performance of the name algorithm, all cases were checked again manually by a Turkish expert to create a 'gold standard'. A more detailed description of the methodology and performance of the name algorithm is published elsewhere [9].

We classified the cancer cases into 12 diagnosis groups (Table 1). Classification was adapted from the International Classification of Childhood Cancer [10]. We then calculated proportional cancer incidence ratios (PCIR) for these diagnosis groups and compared the relative incidence of individual cancers among Turkish and non-Turkish (for the most part German) children. PCIRs compare the proportion of one diagnosis site or group, in relation to all cancer cases. Thus, if 10% of all Turkish cases had brain tumours compared to 5% of all Non-Turkish cases, the PCIR would be 10% divided by 5%, resulting in a PCIR of 2. For PCIRs, confidence intervals can be calculated using established methods [11].

**Table 1:** Classification of cancer cases into 12 diagnosis groups and corresponding ICD-O-2 codes

**Diagnostic group**	**ICD-O-2 codes**
Lymphoid leukaemia	9820–9827, 9850
Acute non-lymphocytic leukemia	9840, 9481, 9861, 9864, 9866, 9867, 9891, 9894, 9910
Hodgkin's disease	9650–9667
Non-Hodgkin/Burkitt Lymphoma	9591–9595, 9670–9686, 9690–9714, 9723, 9687
CNS tumors	9383, 9390–9394, 9380, 9381, 9400–9441, 9470–9473, 9380, 9382, 9384, 9442–9460, 9481, 8270–8281, 8300, 9350–9362, 9480, 9505, 9530–9539, 8000–8004
Neuroblastoma and ganglioneuroblastoma	9490, 9500
Retinoblastoma	9510–9512
Nephroblastoma	8960, 8963, 8964,
Malignant bone tumours	9180–9200, 9220–9230, 9231–9240, 9260, 9363, 9364, 8812, 9250, 9261–9330, 9370, 8000–8004, 8800, 8801, 8803, 8804
Soft-tissue sarcomas	8900–8920, 8991, 8810, 8811, 8813–8833, 9540–9561, 9140, 8840–8896, 8982, 8990, 9040–9044, 9120–9134, 9150–9170, 9251, 9581, 8963, 9231, 9240, 9363, 9364, 9260, 8800–8804
Germ-cell tumors	9060–9102, 8010–8041, 8050–8075, 8082, 8120–8122, 8130–8141, 8143, 8155, 8190–8201, 8210, 8211, 8221–8241, 8244–8246, 8260–8263, 8290, 8310, 8320, 8323, 8430, 8440, 8480–8490, 8504, 8510, 8550, 8560–8573, 8380, 8381, 8441–8473, 8590–8670, 9000, 8000–8004

Other	All other diagnoses including unspecified cancer sites

In the analyses we stratified for age, sex, and year of diagnosis. This was necessary because the number of cases as well as the proportion of Turkish cases differed between 1980 and 2005 due to changes in the completeness of registration and the German reunification in 1990. To test for confounding or interactions we performed a multiple logistic regression using proportional incidence odds ratios with one diagnostic group as 'event' and the other diagnostic groups as 'control' including descent (Turkish versus Non-Turkish), age (in age groups), sex, and time period of diagnosis (in years) as independent variables. Incidence (or risk) ratios, like the PCIR, are in general more conservative than odds ratios due to the larger denominator in incidence ratios. Therefore we calculated the proportional cancer incidence odds ratios in a regression model only to check for confounding or interaction between the independent variables. Here we only show the crude and stratified PCIRs.

We considered different ways to conduct not only proportional analyses but to estimate actual incidence rates. For this purpose we evaluated different approaches to estimate the population under risk for the Turkish cases. However, only population data on children with Turkish nationality in Germany are available, which exclude the naturalised children of Turkish descent. We decided not use these data as this would have led to a substantial overestimation of the true cancer risk of the Turkish cases due to an underestimation of the population at risk.

## Results

**Figure 1:**
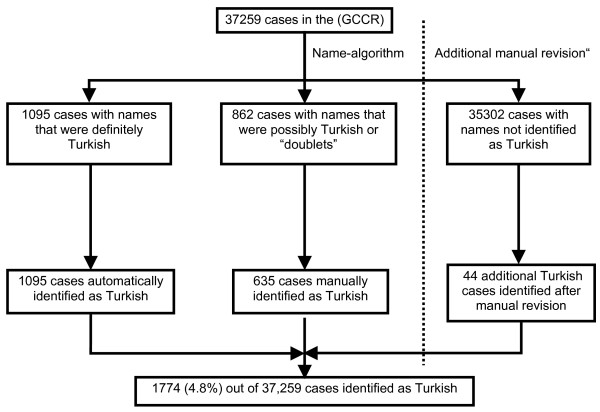
Result of the name based identification of children with Turkish names in the data base of the German Childhood Cancer Registry (GCCR), 1980–2005.

The name algorithm performed well with a high sensitivity and specifity, and we identified 1774 childhood cancer cases of Turkish descent (Figure 1).

The proportion of cases with Turkish names is not dependent on sex or age group; this is true for all cancers as well as specific cancer diagnoses and years of diagnosis. The male-female ratio is 1.42 for cases with Turkish names and 1.27 for the comparison cases.

The PCIRs were close to one for most diagnostic groups. They were significantly above one for acute non-lymphocytic leukemia, Hodgkin's disease and Non-Hodgkin/Burkitt Lymphoma (Table 2). The observation of higher PCIRs for these three diagnostic groups was consistent, but not always statistically significant after stratification for sex (Table 3 &4), and age groups (see Additional file 1). The proportion of retinoblastoma was slightly lower among Turkish cases than among non-Turkish cases after stratification for age; nephroblastoma were significantly less frequent among Turkish children in the age group 5–<10 years (see Additional file 1). The PCIRs did not change in the regression model accounting for sex, age group and year of diagnosis (results of regression model not shown) and no confounding or effect modifications between the independent variables were found. Therefore we present the unadjusted PCIRs.

**Table 2:** Proportional cancer incidence ratios (PCIR) and 95% confidence intervals (95% CI) of children with Turkish names versus children with non-Turkish names in the German Childhood Cancer Registry 1980–2005, both sexes

Diagnosis group	Turkish cases (n)	Non-Turkish cases (n)	PCIR (95% CI)
Lymphoid leukaemia	504	10177	0.99 (0.92–1.07)
Acute non-lymphocytic leukemia	112	1827	**1.23 (1.02–1.47)**
Hodgkin's disease	119	1778	**1.34 (1.13–1.59)**
Non-Hodgkin/Burkitt Lymphoma	152	2553	**1.19 (1.02–1.39)**
CNS tumors	318	6681	0.95 (0.86–1.05)
Neuroblastoma and ganglioneuroblastoma	144	2875	1.00 (0.86–1.17)
Retinoblastoma	28	804	0.70 (0.48–1.01)
Nephroblastoma	92	2167	0.85 (0.70–1.04)
Malignant bone tumours	78	1749	0.89 (0.72–1.11)
Soft-tissue sarcomas	102	2290	0.89 (0.74–1.08)
Germ-cell tumors	48	1142	0.84 (0.64–1.11)
Other	77	1442	1.07 (0.86–1.33)

Total	1774	35485	-

**Table 3:** Proportional cancer incidence ratios (PCIR) and 95% confidence intervals (95% CI) of children with Turkish names versus children with non-Turkish names in the German Childhood Cancer Registry 1980–2005, female children

Diagnosis group	Turkish cases (n)	Non-Turkish cases (n)	PCIR (95% CI)
Lymphoid leukaemia	211	4430	1.02 (0.91–1.14)
Acute non-lymphocytic leukemia	49	870	1.21 (0.92–1.58)
Hodgkin's disease	41	708	1.24 (0.92–1.67)
Non-Hodgkin/Burkitt Lymphoma	44	736	1.28 (0.96–1.70)
CNS tumors	138	2944	1.00 (0.86–1.17)
Neuroblastoma and ganglioneuroblastoma	60	1327	0.97 (0.76–1.23)
Retinoblastoma	14	388	0.77 (0.46–1.30)
Nephroblastoma	42	1119	0.80 (0.60–1.08)
Malignant bone tumours	38	837	0.97 (0.71–1.32)
Soft-tissue sarcomas	36	1032	0.75 (0.54–1.03)
Germ-cell tumors	25	626	0.85 (0.58–1.26)
Other	34	646	1.13 (0.81–1.57)

Total	732	15663	-

**Table 4:** Proportional cancer incidence ratios (PCIR) and 95% confidence intervals (95% CI) of children with Turkish names versus children with non-Turkish names in the German Childhood Cancer Registry 1980–2005, male children

Diagnosis group	Turkish cases (n)	Non-Turkish cases (n)	PCIR (95% CI)
Lymphoid leukaemia	293	5747	0.97 (0.88–1.07)
Acute non-lymphocytic leukemia	63	957	1.25 (0.99–1.59)
Hodgkin's disease	78	1070	**1.39 (1.12–1.72**)
Non-Hodgkin/Burkitt Lymphoma	108	1817	1.13 (0.95–1.35)
CNS tumors	180	3737	0.92 (0.80–1.05)
Neuroblastoma and ganglioneuroblastoma	84	1548	1.03 (0.84–1.27)
Retinoblastoma	14	416	0.64 (0.38–1.08)
Nephroblastoma	50	1048	0.91 (0.69–1.19)
Malignant bone tumours	40	912	0.83 (0.62–1.13)
Soft-tissue sarcomas	66	1258	1.00 (0.79–1.26)
Germ-cell tumors	23	516	0.85 (0.57–1.27)
Other	43	796	1.03 (0.77–1.38)

Total	1042	19822	-

## Discussion

Our study provides insight into the distribution of cancer among Turkish migrant children in Germany. This group of Turkish migrant children defined by their Turkish names stands for a group that has in common a descent from Turkey. Most of these children are the offspring of Turkish migrants who came to Germany after the 1960ies and hence are 2^nd ^generation migrants. The aim of this study was to compare the cancer patterns of this group to the patterns of the indigenous German population. The Turkish names used as indicator here allow drawing conclusions only about the common descent from a country (not the same ethnic group) as well as a common migration experience in the 1^st ^or 2^nd ^generation. This operationalisation of migrant status by descent from a country is commonly used in migrant research. It is, of course, a surrogate for a multidimensional set of factors including genetic, behavioural and contextual variables. The individual measurement of these factors was not possible in our retrospective, registry-based study.

The cancer diagnoses in our study are generally similarly distributed among Turkish and non-Turkish children and there is no evidence that the proportion of Turkish cases differs by age or sex group. For acute non-lymphocytic leukemia, Hodgkin's disease and Non-Hodgkin/Burkitt lymphoma, the proportions are slightly increased for Turkish children. This might be the result of a truly different cancer risk of Turkish children, for which possible causes or causal pathways are not yet known, or due to chance. The confidence intervals presented here are not adjusted for the testing of several subgroups. As we used 12 subgroups, about one spuriously significant result is likely.

Our study has several other limitations. We performed an explorative 'case only' analysis. The PCIR for one diagnosis group is by definition dependent on the PCIRs in the other diagnosis groups as all proportions jointly always have to sum up to 1. The PCIR is therefore not a measure of relative risk and is somewhat difficult to interpret. An increased PCIR for one cancer site could be the expression of lower case frequencies for other cancer sites. Even if the PCIR for one cancer site is increased, the overall cancer risk can still be much lower than in the comparison group.

Misclassification due to inaccurate classification of children with binational parents could also be a cause of bias. Binational marriages between Turkish and German persons have been scarce in the past but are becoming more frequent over the last years. Currently there are about 79,000 Turkish-German marriages in Germany, representing about 10% of all marriages of Turkish persons.

Our findings are internally consistent. The increased PCIRs remained elevated after stratification for sex and age; no confounding by or interactions between these independent variables was found.

As a first step, epidemiologic studies on cancer among migrants such as ours frequently use a descriptive comparative approach and analyse the differences of cancer patterns between migrants and an indigenous/reference population. However, most studies on cancer among migrants focus on adult cancer [12-16], and few studies on childhood cancer are available.

In terms of aetiogical explanations for possible risk differences, migrant children might, besides their possibly different genetic background or different life style, be exposed to different patterns of infections. Concerning acute lymphoid leukaemia, an influence of infectious exposures caused by unusual population mixing, e.g. in heterogeneous and transient populations, is being discussed [17,18]. On the other hand it has been suggested that reduced exposure to infections in very young children may be a risk factor for acute lymphoid leukaemia [19] possibly involving lack of stimulation of the immune system. Migrant children might be a population under particular risk for this cancer due to population mixing or increased contact to infectious agents.

Our study of cancer patterns, however, does not lend support to the hypothesis that migrant children of Turkish descent might have increased risks for acute lymphoid leukemia due to differing patterns of exposure to infectious agents. However, more detailed information is necessary for an in-depth assessment of this issue.

The increased PCIRs for lymphomas in our study are consistent with the findings of studies in migrant populations in other countries. Hemminki et al. found an increased risk for non-Hodgkin lymphoma for children of Turkish parents, especially for those less than 5 years of age [16]. Cummins et al. found an increased risk for lymphoma for South-Asian children in England [20]. The other elevated risks demonstrated in the study by Cummins et al., especially for leukemia, are not in line with our findings.

Because of the small number of Turkish cases for some cancer diagnoses and the resulting limited explanatory power of the respective PCIRs, we grouped the cancer cases in 12 diagnosis groups, including a group 'others'. A detailed analysis of the cancer diagnoses in this latter group showed an increased PCIR for cancer of the nasopharynx among Turkish migrants (PCIR = 2.4, KI = 1.0–5.8, data not shown). This estimate is based on only five Turkish cases and could be a chance finding. However, the result is consistent with the SIR of 8.2 for cancer of the nasopharynx that Visser and Leeuwen found among Turkish migrants in the Netherlands [21]. The increased SIR for cancer of the liver among Turkish migrants (SIR = 4.6) in their study is not supported by our findings, but again the number of Turkish cases with liver cancer in our study is small.

Our data on lymphoma and nasopharyngeal cancer could tentatively be interpreted as supportive of a higher proportion of cancers associated with the Epstein-Barr virus in children with a Turkish name. Indeed, data on childhood cancer from the Izmir registry in Turkey computed through the ACCIS system [22] indicate that EBV-related cancers may be more frequent in Turkey than in Germany. For the period 1993–96, Burkitt lymphoma incidence in Izmir was 4.4 per million children aged 0–14 (World Standard), against 1.1 per million in Germany. However, case numbers are very small and thus need to be interpreted with care. Nevertheless, this may be a point for further in-depth studies.

We were restricted to the 'case only' PCIR analysis, because of the difficulty to define the reference (denominator) population for the children of Turkish descent. The estimation of incidence rates was thus not possible in this study. A valid reference population for the Turkish cases would have been all children of Turkish descent living in Germany in the years 1980–2005. However, such a population estimate is not available. Population registries routinely collect information only on nationality but not on descent. Thus, naturalised children of Turkish descent are no longer identifiable. An earlier effort in the framework of this study to estimate the number of children of Turkish descent in the 'population under risk' using the name algorithm in a representative sample of the population of Germany was not successful due to major changes in the naturalisation law in the study period (1980–2005) and large geographical variances in the proportion of Turkish migrants. In addition, population figures from the past are available only in very few regions of Germany. The large and changing differences between the proportion of Turkish children defined by their names and defined by nationality would introduce a considerable and uncontrollable numerator-denominator bias in estimating incidence rates.

The name-based approach once more proved to be a useful way to identify persons with Turkish descent in Germany. The name algorithm performed well and had high positive and negative predictive values [9]. The approach has some limitations: it can only be used to identify persons of Turkish descent, not migrants of other background and it does not differentiate between the migrant generations. Stratification by country of birth was not possible because in the database of the GCCR, the respective information is incomplete and not validated. Thus a comparison of different immigrant generations (born in Turkey vs. born in Germany) was not possible.

## Conclusion

In the future, harmonized and standardized definitions in European countries, clearly defining who is classified as a migrant and who is not, would be very helpful. A harmonized definition of migrant populations based on a standardized set of variables (1^st^, 2^nd ^Generation, country of birth, ethnicity etc.) would enable a comparable collection of migrant data in health-related and general population data bases and thus allow for linking data from different data sources. Thus further research on migrant health would be facilitated. In the case of childhood cancer, further research should focus on factors influencing the cancer incidence of Turkish children and other migrant or ethnic minority groups, and investigate etiological hypotheses for specific cancers (such as haematological or EBV-related cancers).

## Competing interests

The authors declare that they have no competing interests.

## Authors' contributions

HZ, OR and JS conceived the study. JS coordinated the study, analysed the data and wrote the first draft of the manuscript. CS and PK provided the data and contributed to the data analysis. OR and HZ participated in the design of the study and the data analysis. All authors helped to draft the manuscript and read and approved it in its final form.

## Pre-publication history

The pre-publication history for this paper can be accessed here:



## Supplementary Material

Additional file 1 Proportional cancer incidence ratios (PCIR) and 95% confidence intervals (95% CI) of children with Turkish names versus children with non-Turkish names in the German Childhood Cancer Registry by age groups, 1980–2005. Click here for file

## References

[B1] Statistisches Bundesamt (2006). [Life in Germany. Households, families and health – findings of the Mikrozensus 2005].

[B2] Bundesamt für Migration und Flüchtlinge (2005). [Migration, intergration and asylum in numbers].

[B3] Stiller CA, Parkin DM (1996). Geographic and ethnic variations in the incidence of childhood cancer. Br Med Bull.

[B4] Parkin DM, Bray F, Ferlay J, Pisani P (2005). Global cancer statistics, 2002. CA Cancer J Clin.

[B5] Hemminki K, Li X (2002). Cancer risks in childhood and adolescence among the offspring of immigrants to Sweden. Br J Cancer.

[B6] Kaatsch P (2005). Deutsches Kinderkrebsregister. Eine international angesehene Datenquelle. Deutsches Ärzteblatt.

[B7] Razum O, Zeeb H, Beck K, Becher H, Ziegler H, Stegmaier C (2000). Combining a name algorithm with a capture-recapture method to retrieve cases of Turkish descent in a German population-based cancer registry. Eur J Cancer.

[B8] Razum O, Zeeb H, Akgün S (2001). How useful is a name-based algorithm in health research among Turkish migrants in Germany?. Trop Med Int Health.

[B9] Spallek J, Kaatsch P, Spix C, Ulusoy N, Zeeb H, Razum O (2006). [Name-based identification of cases of Turkish origin in the childhood cancer registry in Mainz]. Gesundheitswesen.

[B10] Steliarova-Foucher E, Stiller C, Lacour B, Kaatsch P (2005). International Classification of Childhood Cancer, Third Edition. Cancer.

[B11] Breslow NE, Day NE (1987). Statistical Methods in Cancer Research – Vol. II – The Design and analysis of cohort studies.

[B12] Parkin DM, Khlat M (1996). Studies of Cancer in Migrants: Rationale and Methodology. Eur J Cancer.

[B13] McCredie M, Williams S, Coates M (1999). Cancer Mortality in East and Southeast Asian migrants to New South Wales, Australia. 1975–1995. Br J Cancer.

[B14] McCredie M, Williams S, Coates M (1999). Cancer Mortality in migrants from the British Isles and continental Europe to New South Wales, Australia. 1975–1995. Int J Cancer.

[B15] Lees S, Papadopoulos I (2000). Cancer and men from minority ethnic groups: an exploration of the literature. Eur J Cancer Care.

[B16] Hemminki K, Li X, Czene K (2002). Cancer risks in First-Generation Immigrants to Sweden. Int J Cancer.

[B17] Kinlen L (2004). Infections and immune factors in cancer: the role of epidemiology. Oncogene.

[B18] Kinlen L (1995). Epidemiological evidence for an infective basis in childhood leukaemia. Cancer.

[B19] Greaves M (1997). Aetiology of acute leukaemia. Lancet.

[B20] Cummins C, Winter H, Maric R, Cheng K, Silcocks P, Varghese C, Battle G (2001). Childhood cancer in the south Asian population of England (1990–1992). Br J Cancer.

[B21] Visser O, van Leeuwen FE (2007). Cancer risk in first generation migrants in North-Holland/Flevoland, The Netherlands, 1995–2004. Eur J Cancer.

[B22] Steliarova-Foucher E, Stiller C, Kaatsch P, Berrino F, Coebergh JW, Lacour B, Parkin M (2004). Geographical patterns and time trends of cancer incidence and survival among children and adolescents in Europe since the 1970s (the ACCISproject): an epidemiological study. Lancet.

