# Supplementary material for: Cancer patterns among children of Turkish descent in Germany: A study at the German Childhood Cancer Registry
Source: BMC Public Health. 2008 May 7;8:152. doi: 10.1186/1471-2458-8-152 (PMC2396623; doi:10.1186/1471-2458-8-152)
Supplement: Additional file 1 — Proportional cancer incidence ratios (PCIR) and 95% confidence intervals (95% CI) of children with Turkish names versus children with non-Turkish names in the German Childhood Cancer Registry by age groups, 1980–2005. [file 1471-2458-8-152-S1.doc]

Proportional cancer incidence ratios (PCIR) and 95% confidence intervals (95% CI) of children with Turkish names versus children with non-Turkish names in the German Childhood Cancer Registry by age groups, 1980-2005

| Age group | 0-<1 years | | | 1-<5 years | | | 5-<10 years | | | 10-<15 years | | |
| --- | --- | --- | --- | --- | --- | --- | --- | --- | --- | --- | --- | --- |
| Diagnosis group | Turkish cases (n) | Non-Turkish cases (n) | PCIR (95% CI) | Turkish cases (n) | Non-Turkish cases (n) | PCIR (95% CI) | Turkish cases (n) | Non-Turkish cases (n) | PCIR (95% CI) | Turkish cases (n) | Non-Turkish cases (n) | PCIR (95% CI) |
| Lymphoid leukaemia | 14 | 273 | 1.09  (0.66-1.80) | 250 | 5100 | 0.93  (0.84-1.02) | 154 | 2976 | 0.98  (0.86-1.11) | 86 | 1828 | 1.06  (0.88-1.28) |
| Acute non-lymphocytic leukemia | 11 | 241 | 0.97  (0.55-1.72) | 33 | 535 | 1.17  (0.84-1.63) | 31 | 452 | 1.30  (0.92-1.82) | 37 | 599 | **1.39**  **(1.02-1.90)** |
| Hodgkin’s disease | - | - | - | 13 | 96 | **2.56**  **(1.50-4.39)** | 52 | 427 | **2.30**  **(1.78-2.98)** | 54 | 1255 | 0.97  (0.76-1.25) |
| Non-Hodgkin/Burkitt Lymphoma | - | - | - | 47 | 465 | **1.91**  **(1.45-2.52)** | 56 | 1015 | 1.04  (0.81-1.33) | 46 | 1023 | 1.01  (0.77-1.33) |
| CNS tumors | 24 | 450 | 1.14  (0.78-1.65) | 121 | 2058 | 1.11  (0.95-1.31) | 107 | 2335 | 0.87  (0.73-1.02) | 66 | 1838 | 0.81  (0.65-1.01) |
| Neuroblastoma and ganglioneuroblastoma | 52 | 1138 | 0.97  (0.78-1.22) | 74 | 1386 | 1.01  (0.81-1.25) | 14 | 272 | 0.97  (0.58-1.63) | - | - | - |
| Retinoblastoma | - | - | - | 17 | 428 | 0.75  (0.47-1.20) | - | - | - | - | - | - |
| Nephroblastoma | 19 | 354 | 1.14  (0.75-1.75) | 57 | 1303 | 0.83  (0.65-1.06) | 12 | 447 | **0.51**  **(0.29-0.89)** | - | - | - |
| Malignant bone tumours | - | - | - | - | - | - | 24 | 450 | 1.01  (0.68-1.49) | 48 | 1178 | 0.92  (0.70-1.20) |
| Soft-tissue sarcomas | - | - | - | 32 | 760 | 0.80  (0.57-1.12) | 29 | 614 | 0.89  (0.63-1.27) | 34 | 692 | 1.11  (0.80-1.53) |
| Germ-cell tumors | 14 | 289 | 1.03  (0.63-1.70) | 12 | 261 | 0.87  (0.50-1.52) | - | - | - | 15 | 409 | 0.83  (0.50-1.36) |
| Other | 13 | 198 | 1.40  (0.83-2.36) | 21 | 402 | 0.99  (0.65-1.51) | 16 | 308 | 0.98  (0.61-1.59) | 27 | 534 | 1.14  (0.79-1.64) |

- PCIRs were not calculated because the number of Turkish cases was smaller than 10.
